# A simple ratio-based approach for power and sample size determination for 2-group comparison using Rasch models

**DOI:** 10.1186/1471-2288-14-87

**Published:** 2014-07-05

**Authors:** Véronique Sébille, Myriam Blanchin, Francis Guillemin, Bruno Falissard, Jean-Benoit Hardouin

**Affiliations:** 1 EA 4275, Biostatistics, Pharmacoepidemiology and Subjective Measures in Health Sciences, University of Nantes, Nantes, France; 2 EA 4360 Apemac, Lorraine University, Paris Descartes University, Nancy, France; 3 INSERM 669, Université Paris-Sud and Université Paris Descartes, Paris, France; 4 AP-HP, Hôpital Paul Brousse, Département de santé publique, Villejuif, France

**Keywords:** Patient-reported outcomes, Item response theory, Rasch model, Sample size, Power

## Abstract

**Background:**

Despite the widespread use of patient-reported Outcomes (PRO) in clinical studies, their design remains a challenge. Justification of study size is hardly provided, especially when a Rasch model is planned for analysing the data in a 2-group comparison study. The classical sample size formula (CLASSIC) for comparing normally distributed endpoints between two groups has shown to be inadequate in this setting (underestimated study sizes). A correction factor (RATIO) has been proposed to reach an adequate sample size from the CLASSIC when a Rasch model is intended to be used for analysis. The objective was to explore the impact of the parameters used for study design on the RATIO and to identify the most relevant to provide a simple method for sample size determination for Rasch modelling.

**Methods:**

A large combination of parameters used for study design was simulated using a Monte Carlo method: variance of the latent trait, group effect, sample size per group, number of items and items difficulty parameters. A linear regression model explaining the RATIO and including all the former parameters as covariates was fitted.

**Results:**

The most relevant parameters explaining the ratio’s variations were the number of items and the variance of the latent trait (R^2^ = 99.4%).

**Conclusions:**

Using the classical sample size formula adjusted with the proposed RATIO can provide a straightforward and reliable formula for sample size computation for 2-group comparison of PRO data using Rasch models.

## Background

Patient-reported outcomes (PRO) are increasingly used in clinical research; they have become essential criteria that have gained major importance especially in chronically ill patients. Consequently, nowadays these outcomes are often considered as main secondary endpoints or even primary endpoints in clinical studies [1-4]. Two main types of analytic strategies are used for PRO data: so-called classical test theory (CTT) and models coming from Item Response Theory (IRT). CTT relies on the observed scores (possibly weighted sum of patients items’ responses) that are assumed to provide a good representation of a “true” score, while IRT relies on an underlying response model relating the items responses to a latent trait, interpreted as the true individual quality of life (QoL) for instance. The potential of IRT models for constructing, validating, and reducing questionnaires and for analyzing PRO data has been regularly underlined [5-7]. IRT and in particular Rasch family models [8] can improve on the classical approach to PRO assessment with advantages that include interval measurements, appropriate management of missing data [9-11] and of possible floor and ceiling effects, comparison of patients across different instruments [12]. Consequently, many questionnaires are validated (or revalidated) using IRT along with CTT [13-15] allowing analysing PRO data with IRT models in clinical research.

Clinical research methodology has reached a high level of requirements through the publication of international guidelines including the CONSORT statement, the STROBE (Strengthening the Reporting of Observational Studies in epidemiology), or TREND (Transparent Reporting of Evaluations with Nonrandomized Designs), initiative for instance [16-19]. All of these published recommendations are aimed at improving the reporting of scientific investigations coming either from randomized clinical trials or observational studies and systematically include an item related to sample size justification and determination. Furthermore, good methodological standards recommend that methods used for sample size planning and for subsequent statistical analysis should be based on similar grounds. Even if guidelines have also been recently published for PRO based studies [20,21], the reporting of such studies often lacks mentioning the justification of study size and its computation. Three main types of situations are often encountered in 2-group comparison studies: i) sample size determination is not performed whatever the intended analysis for PRO data (CTT and/or IRT), ii) tentative justification is occasionally given *a posteriori* for the size of studies, iii) sample size computation is made *a priori* but only relies on CTT (mostly using the classical formula for comparing normally distributed endpoints on expected mean scores) even if IRT models are envisaged for data analysis. In this latter case, previous studies have shown that the classical formula was inadequate for IRT models because it leads to underestimation of the required sample size [22]. From this perspective, a method has been recently developed for power and sample size determination when designing a study using a PRO as a primary endpoint when IRT models coming from the Rasch family are intended to be used for subsequent analysis of the data [23]. This method, named Raschpower, provides the power for a given sample size during the planning stage of a study in the framework of Rasch models. It depends on the following parameters (that are *a priori* assumed and fixed): the parameters related to the items of the questionnaire (items' number J and difficulties parameters δ_j_, j = 1,…,J), the variance of the latent trait (σ^2^) and the mean difference between groups on the latent trait (γ). Some of these parameters are easily known *a priori* when planning a study (e.g. number of items) others are sometimes more difficult to reach (e.g. items difficulties, σ^2^, γ) and initial estimates based on the literature or pilot studies are required. Besides, whether all these parameters have the same importance regarding sample size determination for Rasch models is unknown. The aim of our paper is to explore the relative impact of these parameters on sample size computation and to identify the most relevant to be used during study design for reliable power determination for Rasch models. Our main objective is to provide a simple method for sample size determination when a Rasch model is planned for analysing PRO data in a 2-group comparison study.

## Methods

### The Rasch model

In the Rasch model [8]*,* the responses to the items are modelled as a function of a latent variable representing the so-called ability of a patient measured by the questionnaire (e.g. QoL, anxiety, fatigue…). The latent variable is often considered as a random variable assumed to follow a normal distribution. In this model, each item is characterized by one parameter (δ_j_ for the jth item), named item difficulty because the higher its value, the lower the probability of a positive (favourable) response of the patient to this item regarding the latent trait being measured.

Let us consider that two groups of patients are compared and that a total of N patients have answered a questionnaire containing J binary items. Let X_ij_ be a binary random variable representing the response of patient i to item j with realization x_ij_, θ_i_ be the realization of the latent trait Θ for this patient, and γ the group effect defined as the difference between the means of the latent trait in the two groups.

For each patient, the probability of responding to each item is:

(1) $$\begin{array}{ll} P(X_{\mathrm{ij}}=x_{\mathrm{ij}}|\theta_{i},\delta_{j}) & \;=\frac{\exp\left\{\left(\theta_{i}+g_{i}\gamma -\delta_{j}\right)\;x_{\mathrm{ij}}\right\}}{1+\exp\left(\theta_{i}+g_{i}\gamma -\delta_{j}\right)},\;\\ & i=1,\ldots ,\;N\;\mathrm{and}\;j\;=\;1,\ldots ,\;J \end{array}$$

where δ_j_ represents the difficulty parameter of item j and g_i_ = 0,1 for patients in the first or second group, respectively. The latent variable Θ is usually a random variable following a normal distribution with unknown parameters μ and σ^2^. Marginal maximum likelihood estimation is often used for estimating the parameters of the model.

### Sample size determination in the framework of the Rasch model – The Raschpower method

We assume that we want to design a clinical trial using a given dimension of a PRO (e.g. the Mental Health dimension of the SF-36) as a primary outcome in a two-group cross-sectional study. Let γ (assumed > 0) be the difference between the mean values of the latent trait (e.g. mental health) in the two groups and σ^2^ the common variance of the latent trait in both groups. We assume that the study involves the comparison of the two hypotheses H_0_: γ = 0 against the two-sided alternative H_1_: γ ≠ 0. If we plan to use a Rasch model that includes a group effect γ (Eq 1) to test this null hypothesis on the data that will be gathered during the study with a given power 1-β_R_ and type I error α, determination of the required sample size can be made using an adapted formula that has been implemented in the Raschpower method [23]*.* This method is based on the power of the Wald test of group effect γ for a given sample size and it is briefly described. To perform a Wald test, an estimate Γ of γ is required as well as its standard error. Since we are designing a study, some assumptions are made regarding the expected values of these parameters. More specifically, Γ is set at the assumed value for the group effect, γ, and its standard error is obtained as follows: an expected dataset of the patient’s responses is created conditionally on the planning values that are assumed for the sample size in each group, the group effect γ, the items difficulties δ_j_, and the variance of the latent trait σ^2^. The probabilities and the expected frequencies of all possible response patterns for each group are computed with the statistical model that will be used for analyzing the data that will be gathered during the study: a Rasch model. The variance of the group effect $\hat{V}\mathrm{ar}\left(\hat{\gamma }\right)$ is subsequently estimated using a Rasch model including a group effect with δ_j_ and σ^2^ fixed to their planned expected values.

The power 1-β_R_ is then computed with the following formula:

(2) $$1-\beta_{R}\approx 1-\Phi \left(z_{1-\alpha /2}-\frac{\gamma }{\sqrt{\hat{V}\mathrm{ar}\left(\hat{\gamma }\right)}}\right)$$

where Φ is the cumulative standard normal distribution function and *z*_1 − *α*/2_ the percentiles of the standard normal distribution. 1 − β_R_ is the power of the Wald test of group effect when a Rasch model is used to detect γ at level α. In practice, γ, σ^2^, and the items' difficulties are unknown population parameters and initial estimates based on the literature or pilot studies are required for calculations.

### Relationship between the Raschpower method and the classical formula for manifest normal variables

Using the same notations as before (γ is the group effect and σ^2^ is the common variance of the latent trait for both groups), we can also compute the required sample size per group (N_C0_ for the first group and N_C1_ for the second group) using the classical formula for comparing normally distributed endpoints with a given power 1-β and a type I error α to detect the group effect γ as follows [24]:

(3) $$N_{C0}=\frac{\left(k+1\right)\times \sigma^{2}\times {\left(z_{1-\alpha /2}-z_{1-\beta }\right)}^{\;2}}{k\times \gamma^{2}}$$

Where N_C1_ = k x N_C0_ (when k = 1, the sample sizes are assumed equal in both groups).

The power 1-β for detecting a difference between groups equal to γ with a total sample size of N_C0_ + N_C1_ and a type I error set to α can also be computed as:

(4) $$1-\beta =\Phi \left(\sqrt{\frac{kN_{C0}\times \gamma^{2}}{\left(k+1\right)\times \sigma^{2}}}-z_{1-\alpha /2}\right)$$

**Figure 1:**
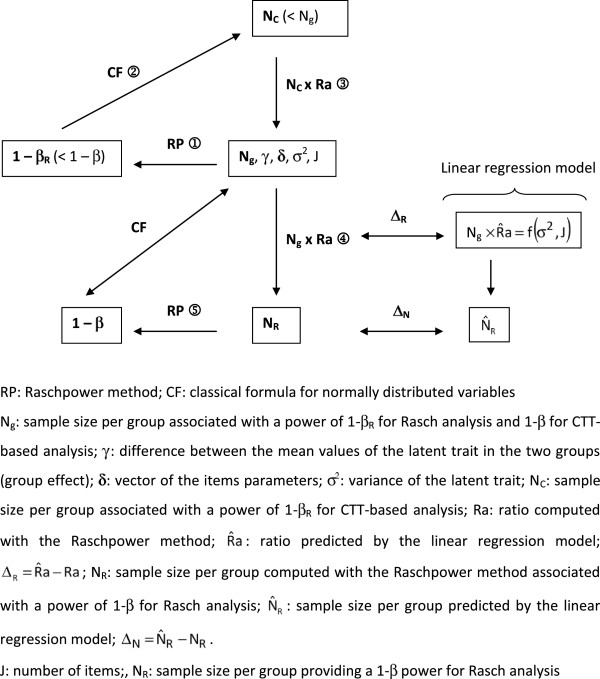
Description of the whole procedure for power and sample size determination using the ratio with the Raschpower method and the linear regression model.

Let us assume without loss of generality that k = 1, that is we expect that the samples sizes are equal in each group (N_C0_ = N_C1_ = N_g_). It has been evidenced [23] that the sample size per group computed using this classical formula (N_g_) allowed obtaining a power of 1-β at level α for CTT-based analysis but did not provide the same power for Rasch-based analysis, but a lower power, computed with the Raschpower method, namely 1-β_R_ ≤ 1-β (Figure 1, RP①). Thus, using this classical formula, the sample size required when a Rasch model is used has to be increased to reach the desired power of 1-β (i.e. N_g_ has to be increased).

It has been observed in a previous study that this increase could be easily computed using the following relationships:

- since 1-β_R_ ≤ 1-β, the sample size that provides a power of 1-β_R_ using the classical formula (Eq 3 and Figure 1, CF②), say N_c_, is lower than N_g_ and the ratio $\mathrm{Ra}=\frac{N_{g}}{N_{c}}$ (Figure 1, ③) is therefore higher than 1

- previous observations [23] have shown that this ratio Ra remained stable for different values of N_g_ and 1-β_R_, given γ, J and items difficulties

- it has been noticed that multiplying N_g_ by this ratio gave a sample size of N_R_ = N_g_ x Ra (Figure 1, ①) that could provide the desired power 1-β for Rasch modelling (Figure 1, RP⑤)

Hence this ratio Ra depends on the well-known classical formula and can be used to provide sample size calculations for Rasch modelling.

### Simulations

A simulation study has been performed in order to get more insight into the relationships between the parameters that are required when planning a study for power determination for a given sample size (γ, σ^2^, δ_j_, J) and the ratio Ra. A large number of cases (10^6^) were simulated with each case corresponding to a single parameter combination (γ, σ^2^, δ_j_, J, N_g_). The parameters values were randomly drawn from continuous or discrete uniform distributions, U[min-max], for: the variance of the latent trait σ^2^ (U[0.25-9]), the group effect γ (U[0.2xσ - 0.8xσ]), the number of items J (U[3-20]), and the sample size per group N_g_ assumed to be equal in both groups (U[50–500]). The items difficulty parameters δ_j_, j = 1,…,J, were drawn from a centred normal distribution with variance σ^2^ and set to the percentiles of the distribution. The Raschpower method was applied on each parameter combination and provided the power 1-β_R_ for Rasch modelling as well as the ratio Ra. Multiple linear regression was performed to assess the contribution of N_g_, γ, J, and σ^2^ and the difficulty parameters δj, j = 1,…,J to the variation of the ratio Ra. The effects of the difficulty parameters on Ra were investigated in several ways for different values of J: i) by introducing each parameter individually δ_j_, j = 1,…,J, ii) by introducing their mean and variance. A two-tailed P-value < 0.05 was considered significant. The variance explained by the model (R^2^) and the root mean square error (RMSE) were obtained and contributed to variable selection. Variables were removed if R^2^ and RMSE remained stable (within a 0.01 range). Post-regression diagnoses were performed to ensure that all linear regression assumptions were met (normality and homoscedasticity of residuals). Statistical analysis was performed using SAS statistical software version 9.3 (SAS Institute Inc, Cary, North Carolina).

## Results

Among the 10^6^ parameter combinations, 15278 corresponded to the largest power for CTT and Rasch-based analysis, 100%, where the ratio cannot be computed. Hence all analyses were performed on 984722 parameter combinations.

A full linear model explaining the value of Ra was first fitted including N_g_, γ, 1/J, 1/σ^2^, the difficulty parameters (included either individually or using their mean and variance) and their interactions. A backward procedure was used for variable selection relying on the R^2^ and RMSE variations between models and not on p-values. Indeed, since the number of simulated combinations was high (984722), all parameters were significant but not necessarily meaningful (very small estimated values). The R^2^ and RMSE remained stable during the backward procedure until the final model only containing 1/J and 1/σ^2^ and their interaction was obtained (a maximum variation of 0.0015 and of 0.0037 was observed for the R^2^ and the RMSE, respectively). The model that was retained can be written as follows:

(5) $$\begin{array}{ll} Ra_{i}= & \beta_{0}+\left(\beta_{1}\times \frac{1}{\sigma_{i}^{2}}\right)+\left(\beta_{2}\times \frac{1}{J_{i}}\right)\\ & +\left(\beta_{3}\times \frac{1}{\sigma_{i}^{2}}\times \frac{1}{J_{i}}\right)+\epsilon_{i} \end{array}$$

where $\epsilon_{i}\sim N\;\left(0,\sigma_{\epsilon }^{2}\right)$, for i = 1, …, 984722

Table 1 shows the estimates of the multiple linear regression model that explains R^2^ = 99.4% of the variance of the ratio and displays high accuracy (RMSE = 0.030). The interaction between 1/σ^2^ and 1/J is significant; the effect of 1/σ^2^ on the ratio seems to be more pronounced when 1/J is large (i.e.: J is small). The ratio increases with 1/σ^2^ (ie: when σ^2^ decreases) and with 1/J (i.e.: when J gets smaller).

**Table 1:** Parameters estimates of the linear regression model explaining the ratio provided by the Raschpower method

**Variables**	**N**_**POP**_ **= 984722**	**P-values**
Intercept	1.012 (7.0 10^−5^)	<10^−3^
1/σ^2^	0.095 (1.0 10^−4^)	<10^−3^
1/J	0.939 (5.0 10^−4^)	<10^−3^
Interaction (1/σ^2^*1/J)	3.730 (7.5 10^−4^)	<10^−3^
R^2^	0.994	/
RMSE	0.030	/

Standard errors in parentheses. σ^2^: variance of the latent trait; J: number of items.

**Figure 2:**
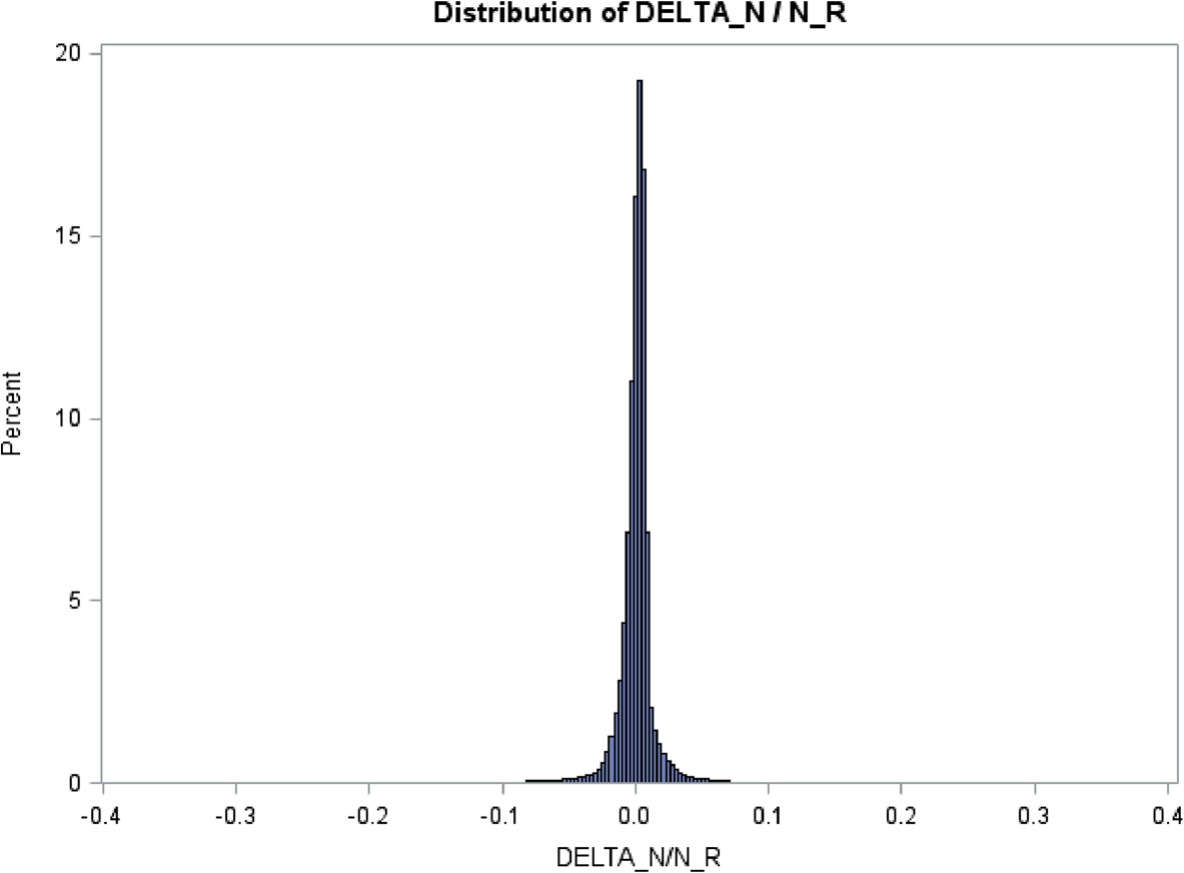
**Distributions of Δ**_**N **_**/ N**_**R **_**with**$\Delta_{N}={\hat{N}}_{R}-N_{R}$**, where**${\hat{N}}_{R}$**is the number of subjects per group predicted by the linear regression model and N**_**R **_**is the number of subjects per group associated with the Raschpower method.**

The number of subjects per group predicted by this model was computed as follows: ${\hat{N}}_{R}=N_{g}\times \hat{R}a$ where $\hat{R}a$ is the ratio predicted by the model. It was compared to the expected number of subject per group N_R_ = N_g_ × Ra where Ra is the ratio derived from the Raschpower method. The difference between the ratio (respectively number of subjects per group) predicted by the model $\hat{R}a$ (respectively ${\hat{N}}_{R}$) and the one associated with the Raschpower method Ra (respectively N_R_) was computed for all parameters combinations with $\Delta_{R}=\hat{R}a-\mathrm{Ra}$ and $\Delta_{N}={\hat{N}}_{R}-N_{R}$. Figure 2 shows the distributions of Δ_N_ / N_R_ which is distributed around 0.

To quantify more precisely the magnitude of the difference Δ_N_, a threshold (Thres) corresponding to 5% of the number of subjects per group expected with the Raschpower method, Thres = 0.05 × N_R_, was calculated for all parameters combinations. The descriptive statistics related to the distributions of Δ_R_, Δ_N_, and to Δ_N_ with respect to Thres are displayed in Table 2. Ninety-five percent of the values of Δ_R_, (respectively Δ_N_) lie between −0.049 and 0.043 (respectively −10.623 and 13.499). The largest overestimation (respectively underestimation) of the number of subjects per group predicted by the model is about 112 subjects per group (respectively −180 subjects per group). The distribution of Δ_N_ mostly lies (98.34% of the cases) within the interval [−Thres – + Thres] corresponding to ±5% of the number of subjects per group expected with the Raschpower method. Moreover, the model rarely predicted (0.56%) an overestimated number of subjects per group of more than 5% of the sample size per group expected with Raschpower. This case only occurs when the variance of the latent trait σ^2^ < 1 and J > 7 items. An underestimated number of subjects per group of more than 5% of the sample size per group expected with Raschpower is occasionally observed (1.10%) and it mostly occurs (more than 90% of the cases) when J is larger than 16 items and mostly when σ^2^ < 1 (75% of the cases).The whole procedure including the Raschpower method and the linear regression model for power and sample size determination using the ratio is summarized in Figure 1.

**Table 2:** **Distributions of the difference between the ratio (respectively number of subjects per group) predicted by the model and the one expected by the Raschpower method Δ**_**R **_**(respectively Δ**_**N**_**) and according to the threshold (Thres) for Δ**_**N**_

**Variables**	**N**_**POP**_ **= 1996077**
	**2.5% / Median / 97.5%**
	**[min-max]**
Δ_R_	−0.049 / 0.002 / 0.043
	[−1.236 ; 0.230]
Δ_N_	−10.623 / 0.438 / 13.499
	[−179.576 ; 112.064]
	**n (%)**
– Thres < Δ_N_ < + Thres	968364 (98.34%)
Δ_N_ < − Thres	10865 (1.10%)^§^
Δ_N_ > + Thres	5493 (0.56%)^†^

Thres : threshold corresponding to 5% of the number of subjects per group derived from the Raschpower method.

^§^: underestimation of the number of subjects per group produced by the model as compared to the Raschpower method; ^†^: overestimation of the number of subjects per group produced by the model as compared to the Raschpower method.

## An example of sample size determination in clinical research using the ratio – NHP data

The data come from a pilot study whose main objective is to compare the pain level of two groups of patients having either Steinert's disease or another muscular dystrophy. The two disease groups have similar symptoms but also present a number of dissimilar features such as pain, cognitive disorders or male hypogonadism that are more frequently encountered in patients suffering from Steinert's disease and may impact QoL. Since QoL and in particular pain assessment may help to better understand the burden of disease from the patients' perspective and improving health outcomes and management, the pain dimension of the Nottingham Health Profile (NHP) questionnaire was used; it is composed of eight binary items (J = 8). The ethics committee of Reims, France granted approval for the study and patients were recruited in the university hospital of Reims: 52 patients were included with Steinert’s disease and 95 patients with others muscular dystrophies. A Rasch model including a group effect γ was fitted on these data and its global fit was not rejected by the R_1m_ test (p = 0.329) [25]. The estimation of the difference between the means of the latent trait of the two groups was $\hat{\gamma }$ = 0.649 and the estimated latent trait's variance was ${\hat{\sigma }}^{2}$= 3.9323 (non-significant difference between groups: p = 0.08). The objective was to use this pilot study to help planning a future possibly larger study that would provide enough power to detect this difference on the latent trait using a Rasch model. Indeed, it seemed valuable to the clinicians to determine a sample size large enough to be able to significantly detect this difference considered as clinically relevant with a power of 1-β = 90% using a Rasch model. The sample size per group computed using the classical formula (Eq 3), for detecting *γ* = 0.649 with a 90% power at α = 5%, assuming *σ*^2^= 3.9323, is N_g_ = 197 for CTT-based analysis. We know that N_g_ has to be increased to reach the desired power for Rasch modelling using the ratio. The ratio predicted by the multiple linear regression model can be easily computed as follows using the values of J and *σ*^2^:

(6) $$\begin{array}{ll} \hat{R}a= & 1.012+\left(0.095\times \frac{1}{3.9323}\right)\\ & +\left(0.939\times \frac{1}{8}\right)+\left(3.730\times \frac{1}{3.9323}\times \frac{1}{8}\right)\\ & =1.27210 \end{array}$$

Multiplying N_g_ by this ratio gives a sample size of ${\hat{N}}_{R}$= 197 × 1.27210 ≈ 251 patients per group that should provide the desired power of 90% for Rasch modelling of the pain dimension of the NHP questionnaire. These results were compared to those obtained with the Raschpower method using the estimated difficulty parameters from the pilot study (2.61, 2.94, 1.75, 0.46, − 0.11, 0.36, 1.28, 2.23), $\hat{\gamma }$ = 0.649, ${\hat{\sigma }}^{2}$= 3.9323, and N_g_ = 197 per group. An 80% power (1-β_R_) is expected using the Raschpower method for Rasch modelling with a sample size of N_g_ = 197 per group (Figure 1, RP①). The proposed ratio is therefore equal to 197 / 147 = 1.34 where N_c_ = 147 is the number of subjects per group that provides a power of 80% using the classical formula (Figure 1, CF②). Hence, using the ratio, 197 × 1.34 ≈ 264 (N_R_) patients per group should provide the desired power of 90% for Rasch modelling (Figure 1, RP⑤).

The parameters that are required for the determination of the ratio using the linear regression model or the Raschpower method as well as their corresponding values appear in Table 3. The ratio provided by the linear regression model and Raschpower (Table 3) are close to one another (Δ_R_ = −0.0679) and the number of subjects per group are |Δ_N_| = 13 patients apart. Moreover, since |Δ_N_| / N_R_ = 13 / 264 = 0.0492, the linear model's prediction was within 5% of the expected sample size provided by the Raschpower method.

**Table 3:** Comparison of the required parameters and the results obtained using the linear regression model and the Raschpower method on the NHP data

**Variables**	**Linear regression model**	**Raschpower method**
σ^2^	3.9323	3.9323
J	8	8
γ	/	0.649
N_g_	/	197
**δ**	/	(2.61, 2.94, 1.75, 0.46, −0.11, 0.36, 1.28, 2.23)
Ra	1.27210	1.34
N_R_	251	264

σ^2^: variance of the latent trait; J: number of items; γ: difference between the mean values of the latent trait in the two groups (group effect); N_g_: sample size per group providing a 1-β = 90% power with the classical formula and a 1-β_R_ = 80% power with Raschpower; **δ**: vector of the items parameters (δ_1_, δ_2_, δ_3_, δ_4_, δ_5_, δ_6_, δ_7_, δ_8_); Ra: ratio, N_R_: sample size per group providing a 1-β ≈ 90% power for Rasch analysis with the linear regression model and Raschpower, /: not required.

## Discussion

**Figure 3:**
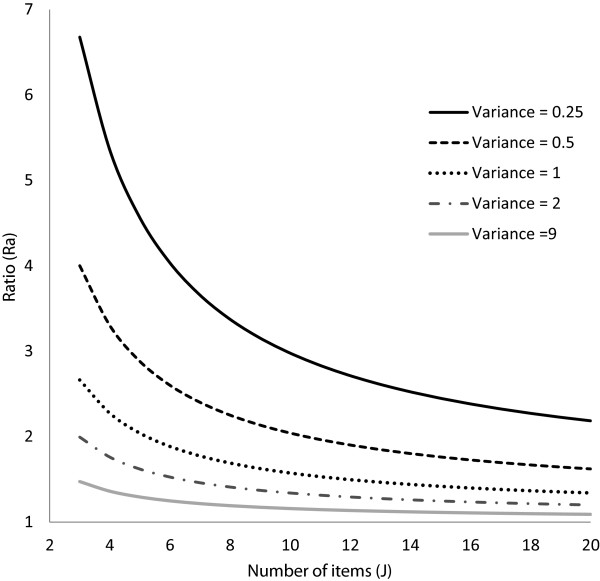
Values of the ratio Ra as a function of the number of items J according to the values of the variance of the latent trait.

Our results revealed that the sample size required in the framework of two-group cross-sectional studies for subsequent use of a Rasch model to analyse PRO data can be easily computed using the classical formula for comparing normally distributed endpoints along with a correction factor (named ratio in this paper). The most relevant parameters explaining this ratio’s variation (R^2^ = 99.4%) were the number of items of the questionnaire to be used in the study (J) and the latent trait’s variance (σ^2^). Hence when designing a study, the most important parameters for reliable power determination using this ratio when a Rasch model is intended to be used to analyse PRO data appear to be the variance of the latent trait and the number of items regardless of the values of the group effect (γ) and items parameters (δ_j_, j = 1,…,J). A preliminary investigation had already evidenced that the precision with which item difficulty parameters were known did not have an impact on power determination of the test of group effect using a Rasch model [22]. However, in this previous study, the number of items J greatly impacted power as it was observed in our current study for sample size determination; both (sample size and power) being very closely related. The power increased with J in line with what we observed in this study where the ratio decreased when J rose from 3 to 20 items, implying that fewer subjects were needed to obtain the same power when J = 20 as compared to J = 3. Moreover, this decrease of the ratio was more marked as σ^2^ got smaller (significant interaction between 1/J and 1/σ^2^). Quite a large range of values were chosen for the variance of the latent trait (from 0.25 to 9) and for the number of items J (from 3 to 20) that allowed investigating more in depth the magnitude of their impact on the ratio. Figure 3 shows the evolution of the ratio Ra as a function of the number of items J according to the values of the variance of the latent trait σ^2^. The effect of σ^2^ on the ratio was large, especially for small values of the variance (σ^2^ < 1), the ratio increasing as σ^2^ decreased. This result, which might be thought as counter-intuitive, comes from the fact that the ratio, used to correct the sample size coming from the classical formula to obtain an adequately powered Rasch model, is a measure of the distance between the sample sizes corresponding to the powers expected for CTT and Rasch-based analyses. This distance becomes larger as the variance gets smaller and it reaches its maximum when σ^2^ < 1. Hence, the correction factor (ratio) is likely to get larger as σ^2^ decreases and the distance between the sample sizes for CTT and Rasch increases. Furthermore, it can be noted that when σ^2^ < 1, the linear regression model could predict an overestimated number of subjects per group of more than 5% of the sample size per group expected with Raschpower (in at most 0.56% of all parameters combinations). An underestimation of more than 5% of the sample size per group expected with Raschpower could also be noticed (in at most 1.10% of all parameters combinations) for small values of σ^2^ (σ^2^ < 1 in 75% of the cases) and large values of J (J > 17 in more than 90% of the cases). It can be emphasized that such small variances for the latent trait might be rarely encountered in practice especially when J is large [26,27]; hence this simple regression model should be reliable for sample size determination in most situations usually found in clinical research. Nevertheless, one of the major issues regarding study design and sample size determination still remains: to what expected values should we fix the key parameters? In our case, the challenge is put on one single parameter, the expected value for the variance of the latent trait. Retrospective, pilot data or published studies can be used for that purpose to provide information regarding the plausible range of values for the variance. However, it can turn out to be problematic if no previous studies can provide this information and it seems important to further study the impact of misspecifications of the planning values for the variance on the performance of the proposed method for sample size determination for Rasch modelling.

The fact that the number of subjects given by the classical formula, based on the latent trait, has to be increased using the ratio to reach the expected power for Rasch modelling could deliver a wrong message. Indeed, it could be interpreted as if Rasch models required more subjects than CTT-based analyses would. In fact, the classical formula is directly computed from the expected difference between the latent traits in both groups and the latent trait's variance in each group, assumed to be equal. By doing so, we assume that the means and variance of the latent traits are "perfectly" known and thus do not take into account the fact that the latent trait is not an observed (manifest) variable. Hence, its estimation requires the use of a model which creates uncertainty, unlike scores that can be directly observed and measured. This uncertainty is taken into account by adjusting the sample size using the ratio to obtain an adequately sized study for Rasch modelling. Moreover, it has been underlined that the so-called effect size (difference in means over the standard deviation) on the score scale was lower than the corresponding effect size on the latent trait scale. Consequently, the sample size requested for CTT-based analysis using the effect size on the score scale is higher than its counterpart on the latent trait scale.

The proposed method can be used with confidence when J stands between 3 and 20 and especially when the variance of the latent trait is expected to be higher than 1. Otherwise (when σ^2^ < 1), the Raschpower method should be preferred since the ratio-based approach might under or overestimate the sample size. One of the limitations of our study is that we focused on one of the most well-known IRT model, the Rasch model. The Raschpower method has also been developed for other models that are well suited for the analysis of polytomous item responses, such as the Partial Credit Model or the Rating Scale Model (Hardouin, under revision). Moreover, the Raschpower method has recently been extended to deal with longitudinal designs [28] and it might be expected that this ratio would also be worthwhile in these contexts. Finally, the Raschpower method (for dichotomous and polytomous items and for cross-sectional and longitudinal designs) and the ratio-based approach (for dichotomous items) have been implemented in the free Raschpower module available at the website PRO-online http://pro-online.univ-nantes.fr.

## Conclusion

Using the classical formula for normally distributed endpoints along with the proposed ratio only depending on the number of items and the variance of the latent trait can provide a straightforward and reliable formula for sample size computation for subsequent Rasch-based analysis of PRO data.

## Competing interests

The authors declare that they have no competing interests.

## Authors’ contributions

VS, JBH and MB have made substantial contributions to conception and design, analysis and interpretation of data; BF and FG has been involved in drafting the manuscript and revising it critically for important intellectual content. All authors read and approved the final manuscript.

## Pre-publication history

The pre-publication history for this paper can be accessed here:

http://www.biomedcentral.com/1471-2288/14/87/prepub
